# Attogram mass sensing based on silicon microbeam resonators

**DOI:** 10.1038/srep46660

**Published:** 2017-04-21

**Authors:** In-Bok Baek, Sangwon Byun, Bong Kuk Lee, Jin-Hwa Ryu, Yarkyeon Kim, Yong Sun Yoon, Won Ik Jang, Seongjae Lee, Han Young Yu

**Affiliations:** 1 Department of Physics, Research Institute for Natural Sciences, Hanyang University, 222 Wangsimri-ro, Seongdonggu, Seoul, 04763, Korea; 2 Bio-Medical IT Convergence Research Department, Electronics and Telecommunications Research Institute (ETRI), 218 Gajeongno, Yuseong, Daejeon, 34129, Korea; 3 Department of Electronics Engineering, Incheon National University, 119 Academy-ro, Yeonsu-gu, Incheon, 22012, Korea

## Abstract

Using doubly-clamped silicon (Si) microbeam resonators, we demonstrate sub-attogram per Hertz (ag/Hz) mass sensitivity, which is extremely high sensitivity achieved by micro-scale MEMS mass sensors. We also characterize unusual buckling phenomena of the resonators. The thin-film based resonator is composed of a Si microbeam surrounded by silicon nitride (SiN) anchors, which significantly improve performance by providing fixation on the microbeam and stabilizing oscillating motion. Here, we introduce two fabrication techniques to further improve the mass sensitivity. First, we minimize surface stress by depositing a sacrificial SiN layer, which prevents damage on the Si microbeam. Second, we modify anchor structure to find optimal design that allows the microbeam to oscillate in quasi-one dimensional mode while achieving high quality factor. Mass loading is conducted by depositing Au/Ti thin films on the local area of the microbeam surface. Using sequential mass loading, we test effects of changing beam dimensions, position of mass loading, and distribution of a metal film on the mass sensitivity. In addition, we demonstrate that microbeams suffer local micro-buckling and global buckling by excessive mass loading, which are induced by two different mechanisms. We also find that the critical buckling length is increased by additional support from the anchors.

Silicon-based resonators fabricated by micro- and nanoelectromechanical systems (MEMS/NEMS) technology have been rapidly developed for a wide range of gravimetric sensing applications, such as force, mass, gas, humidity, infrared (IR) thermal, and biomolecular sensors^1^,^2^,^3^,^4^,^5^,^6^, based on high sensitivity and resolution for mass detection^7^,^8^. To achieve highly sensitive mass detection, low effective mass, high spring constant, mediated high resonance frequency, and high quality factor (Q-factor) are required. In particular, various approaches have been used to increase the Q-factor, including reduction in resonator size to the nm scale, reduction in effective mass, and optimization of anchor structure^9^,^10^,^11^. In general, dissipation of mechanical energy during oscillation deteriorates the Q-factor. Anchor loss (also called clamping loss or support loss) in vacuum, which occurs between the beam and supporting clamp, causes most significant loss of energy compared with other dissipation mechanisms, such as acoustic and viscous losses^12^,^13^. Therefore, the loss of energy can be most effectively reduced by preventing the anchor loss, which can be achieved by optimizing dimensions of the resonator. To reduce the anchor loss, previous studies have utilized numerical simulation and suggested modified structural design^14^,^15^. However, since the anchor and beam are often formed together on the same substrate, both structures are exposed to damage and suffer deformation during fabrication procedure, which causes the final shape of the resonator to deviate from its original design. This causes increase in energy flow through the anchor, leading to decrease in the Q-factor. Deformation also increases non-linearity and asymmetry of oscillating motion, leading to fluctuation in resonance frequency^16^,^17^.

The NEMS sensors attracted much interest due to small effective mass and high surface-to-volume ratio although they have adopted the same designs and fabrication processes of the MEMS sensors. The NEMS sensors have enabled highly sensitive mass detection but their dynamic range decreases with beam dimensions, which significantly limits their availability^18^. In addition, an elaborate measurement system is required to differentiate vibrating motion caused by diffraction and background noise due to reduced beam size^19^,^20^,^21^,^22^,^23^.

In contrast, thin film-based MEMS resonators, which can be mass-produced at low cost, have demonstrated a wider dynamic range while maintaining high sensitivity due to large surface area and low effective mass. However, the large surface area can induce higher surface stress on the thin film when the resonator is subjected to compression, bending, shear, or combination of these stress factors. Large area can also increase chance of having imperfection on the surface structure during fabrication procedure, such as defects and geometric irregularities^24^,^25^. Large surface stress and imperfection can cause increases in non-linearity and energy dissipation in oscillating motion, which impair resonator’s capacity as a mass sensor. Therefore, design of beam structure and fabrication procedure for the thin film-based MEMS resonator should take the surface stress and imperfection into consideration^26^,^27^.

In addition, excessive increase in these stress factors on the resonator surface results in buckling. Buckling of a plate-type structure is typically classified into three modes, global, local, and lateral-torsional buckling^28^,^29^. Global buckling is associated with the entire plate deforming in out-of-plane direction due to uniform compression. Local buckling forms one or multiple sinusoidal waves on the plate due to uneven local compression. Lateral-torsional buckling is characterized by lateral deflection and torsional rotation of the entire plate. Critical buckling stress and buckling mode are significantly affected by geometry of the structure. Therefore, modifying beam dimensions, such as thickness-to-length ratio, can reduce the chance of buckling but it can also affect the performance of the resonator. For example, a resonator with a thicker beam is less susceptible to buckling but it requires higher bias voltage to operate, which increases thermal noise and also complicates integration with other electronic elements in the system.

To overcome these limitations, we have recently developed a thin-film based doubly-clamped Si microbeam resonator surrounded by SiN anchors, which significantly improved the performance of the resonator without modifying the geometry of the microbeam^30^. Since the surrounding structure was formed by SiN and silicon dioxide (SiO_2_) instead of Si, we were able to achieve higher selectivity during fabrication. This change minimized undesirable deformation of structures during fabrication, which reduced fluctuation in resonance frequency. Less deformation also allowed us to fabricate the resonator as we originally designed, which minimized structural asymmetry of the Si microbeam. The SiN anchors stabilized oscillating motion at the anchor, leading to reduced anchor loss, and eliminated harmonic oscillations, which were observed from resonators without the anchors. Resulting oscillation appeared as a single normal mode (quasi-one dimensional) with significantly improved Q-factor.

Here, we demonstrate that the Si microbeam resonator surrounded by the SiN anchors can operate as a mass sensor capable of highly sensitive detection at the ag/Hz level. To further improve the performance of the resonator, we modify fabrication procedure to reduce surface stress and imperfection, and to optimize the design of anchor structure. We demonstrate that our method can produce resonator sensors with significantly improved Q-factor (~30000) and mass sensitivity (~1 ag/Hz). Effects of beam dimensions, position of mass loading, and distribution of a loaded mass on the mass sensitivity are characterized. Furthermore, buckling of microbeams caused by overloading is studied to understand failure of the mass sensor. We observe two types of buckling, global and local micro-buckling, leading to different changes in beam shape and mass sensing characteristic. In particular, unlike the conventional type of local buckling as described above, the local micro-buckling reported in this study only affects the area where the mass is loaded, resulting in local bend. We also show that the SiN anchors can increase critical buckling length by comparing theoretical model of critical buckling stress with experimental results.

## Results

**Figure 1:**
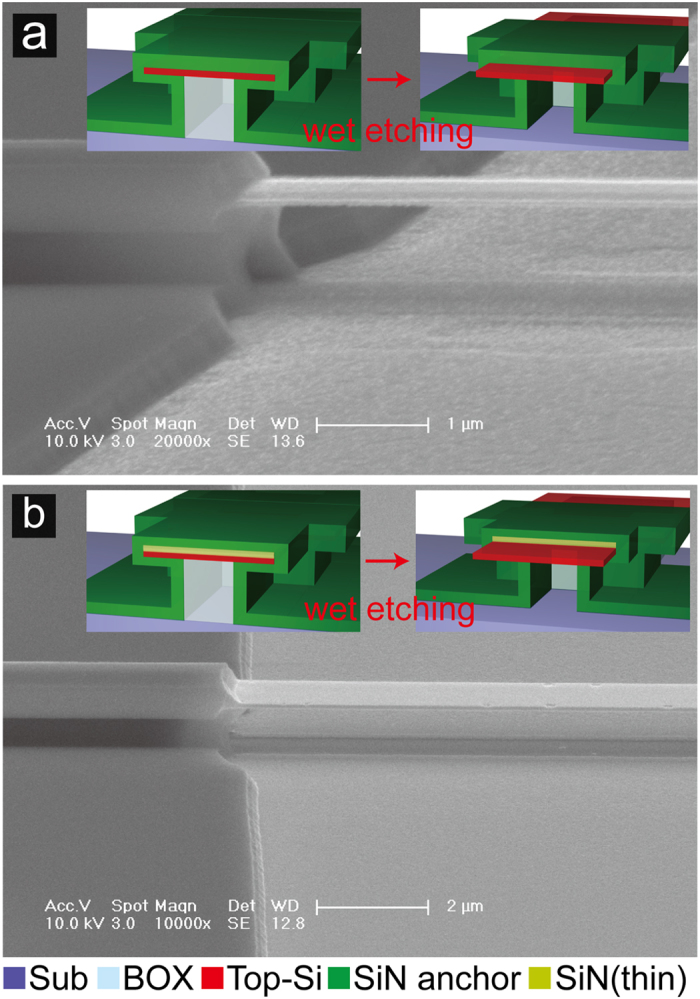
Effect of the SiN sacrificial layer on Si microbeam surface during wet etching. SEM images of resonator anchors, which are fabricated (**a**) without and (**b**) with the SiN sacrificial layer. Insets show cross-sectional schematic illustrations of the resonator anchors fabricated without and with the SiN sacrificial layer.

To reduce the surface stress on the microbeam, we have modified the fabrication procedure to include deposition of a 15 nm thick sacrificial SiN layer on the top Si layer by low-pressure chemical-vapor-deposition (LPCVD) at 750 °C (Figure S1 in the Supporting Information). Figure 1a and bshow scanning electron microscopy (SEM) images of the Si microbeam surfaces fabricated without and with the SiN sacrificial layer. Schematic illustrations of two different microbeam structures are shown in insets. Without deposition of the sacrificial layer, the microbeam was damaged by wet etching which was conducted to form SiN anchor structure, resulting in non-uniform beam thickness and increase in surface roughness (Fig. 1a). In contrast, the microbeam fabricated with the sacrificial layer showed more uniform thickness and clearer surface, demonstrating that the sacrificial layer protected the microbeam surface during wet etching (Fig. 1b).

The SiN layer was etched in heated phosphoric acid solution at 160 °C based on its etching selectivity to SiO_2_. However, SiN layers on the undercut area and on the microbeam surface were not etched at the same rate, which resulted in either damaged beam surface due to excessive exposure to etchant or residual of the sacrificial layer left on the microbeam surface due to insufficient etching time. Since the residual sacrificial layer can affect resonant properties of the microbeam, we tested resonators with the residual, which indeed showed significantly lower resonance frequency and Q-factor than those without the residual (Figure S2). Furthermore, resonators with the residual required higher actuation voltage in linear region of frequency response (Figure S3). To eliminate the problem, we optimized the thickness of the sacrificial layer, which was 15 nm in the current study, to ensure clean removal of the sacrificial layer without damaging the microbeam surface. As a result, we were able to demonstrate that the performance of resonators can be improved by depositing the SiN sacrificial layer, which reduces non-uniformity in thickness and surface roughness.

**Figure 2:**
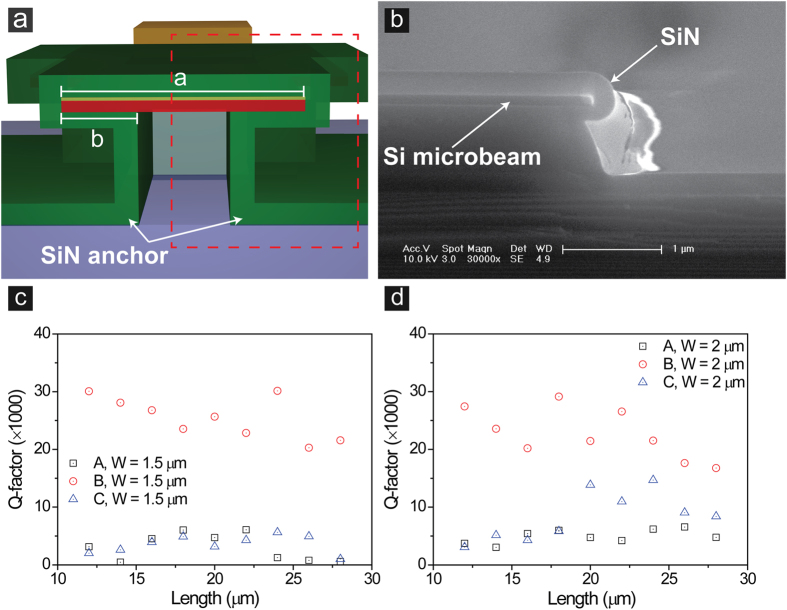
Modifying coverage ratio of SiN anchors. (**a**) Cross-sectional schematic illustration and (**b**) a SEM image of the Si microbeam surrounded by the SiN anchor. Effect of increasing coverage ratio on Q-factor measured from (**c**) 1.5 μm wide and (**d**) 2 μm wide microbeams with various beam lengths (A = 35.9%, B = 56%, and C = 96.1% in c; A = 26.9%, B = 42%, and C = 72 in d). Coverage ratio (%) = ((2 × b)/a) × 100, where a is the beam width and b is the width of undercut area.

To optimize oscillating motion of the doubly-clamped resonator, we modified coverage ratio of the SiN anchor structure. Isotropic wet etching on the undercut area was controlled to define coverage ratios, which were later estimated from SEM images of microbeams (Fig. 2a and b, and Figure S4). The coverage ratio is the ratio of the width of the undercut area to the total width of the microbeam. The anchors provide well-defined boundary conditions at both ends of the beam, which can help the microbeam to oscillate like an ideal 1-D structure while avoiding bending or torsional motion. To verify covering effect of the SiN anchors, mechanical responses of resonators to frequency-mediated electric field were measured (Fig. 2c and d). Length of microbeam was varied from 12 to 28 μm and two different widths (1.5 μm, 2 μm) were tested. Coverage ratios were increased from 36 to 96% and 27 to 72% for resonators of 1.5 μm and 2 μm widths, respectively. Table 1 summarizes dimensions of microbeams (width × length), their coverage ratios, and highest Q-factors measured from each sample. Samples B, which had higher coverage ratio than Samples A, also showed higher Q-factors (>30000). However, although Samples C had the highest coverage ratios, Q-factors were lower than those of Samples B since the microbeam was bent-up at the undercut area (Figure S4c). These results suggest that the Q-factor can be improved by increasing the coverage ratio provided that the microbeam is maintained flat during fabrication procedure. We were able to increase the Q-factor by a factor of at least five by altering the coverage ratio.

**Figure 3:**
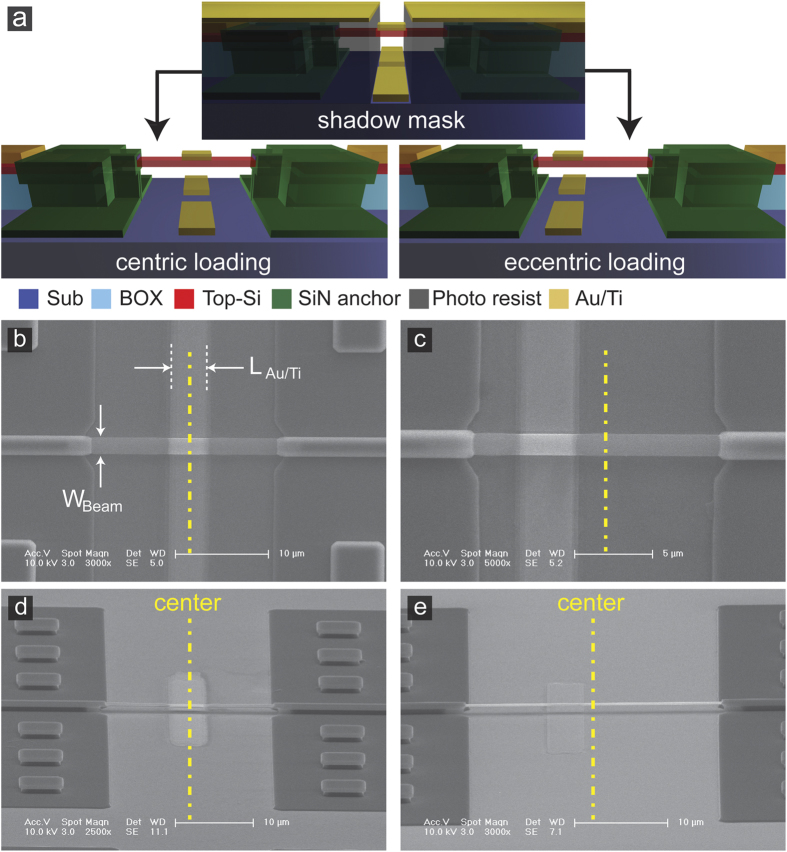
Mass loading on Si microbeam surface by depositing Au/Ti thin films. (**a**) Schematic diagrams of the lift-off process for mass loading on the center (centric loading) and off-center (eccentric loading). Top-view SEM images of the 3.5 μm long Au/Ti thin film loaded (**b**) on the center and (**c**) off center (L_Au/Ti_ = 3.5 μm). Perspective-view SEM images of the 4 μm long Au/Ti thin film loaded (**d**) on the center and (**e**) off center (L_Au/Ti_ = 4 μm).

To test mass detection of the resonator, we measured resonance frequency shift from sequential mass loading on the microbeam. Mass loading was performed by depositing Au/Ti thin films on the local area of the microbeam surface using electron beam evaporation and a lift-off process (Fig. 3). Electron beam evaporation has been widely used to deposit uniform thin films based on their low thermal stress under high vacuum condition. The lift-off process can reliably produce undamaged microbeam surface using protection from a photoresist layer. The photoresist layer can also prevent buckling of microbeams by immobilizing their structures during metal deposition. After deposition was completed, the thin metal film on the microbeam was obtained by immersing the device in acetone solution at room temperature. Schematic diagrams of the metal evaporation process and Au/Ti thin film loading on the center and the off-center are shown in Fig. 3a. SEM images of the resulting Au/Ti thin films on both centric and eccentric loading positions are shown in Fig. 3b−e. The lengths of the metal film (L_Au/Ti_) were 3.5 μm for microbeams in Fig. 3b and c, and 4 μm for those in Fig. 3d and e. The thicknesses of Ti and Au were 2 nm and 4 nm, respectively, which were measured by atomic force microscopy (AFM).

**Figure 4:**
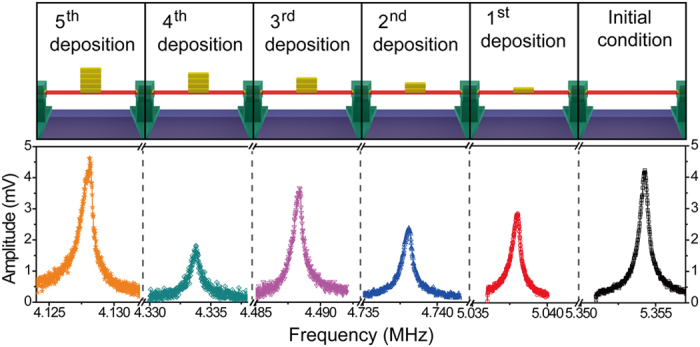
Resonance frequency shifts from sequential mass loading on the Si microbeam. (top) Schematic illustration of sequential mass loading. (bottom) Resonance frequency moves toward lower frequency band as the mass increases. Colors indicate deposition sequences (black: initial condition, red: first deposition, blue: second deposition, magenta: third deposition, dark cyan: fourth deposition, orange: fifth deposition).

Resonance frequency shifts from sequential mass loading were measured in vacuum condition (~1 mTorr). As the mass increased, the resonance peak moved toward lower frequency band but the full width at half maximum (*Δf*) and Lorentzian peak remained similar (Fig. 4). Resonant parameters, such as resonance frequency, full width at half maximum, peak-to-peak voltage, and quality factor, measured from all mass loading conditions are summarized in Table 2. The peak value and the quality factor varied depending on loading conditions. Fluctuations in those parameters were partially caused by changes in laser alignment or scattering, which are known to affect the reflectance of the laser from the microbeam surface. For instance, the second mass loading condition showed a lower quality factor than the third loading. However, the difference in the quality factor between those two measurements was not expected to significantly affect the resonant properties. Actuation voltage for oscillation significantly increased after the fifth deposition, suggesting a sudden change in physical property of the resonator, such as buckling of the microbeam, which will be discussed in detail later.

**Figure 5:**
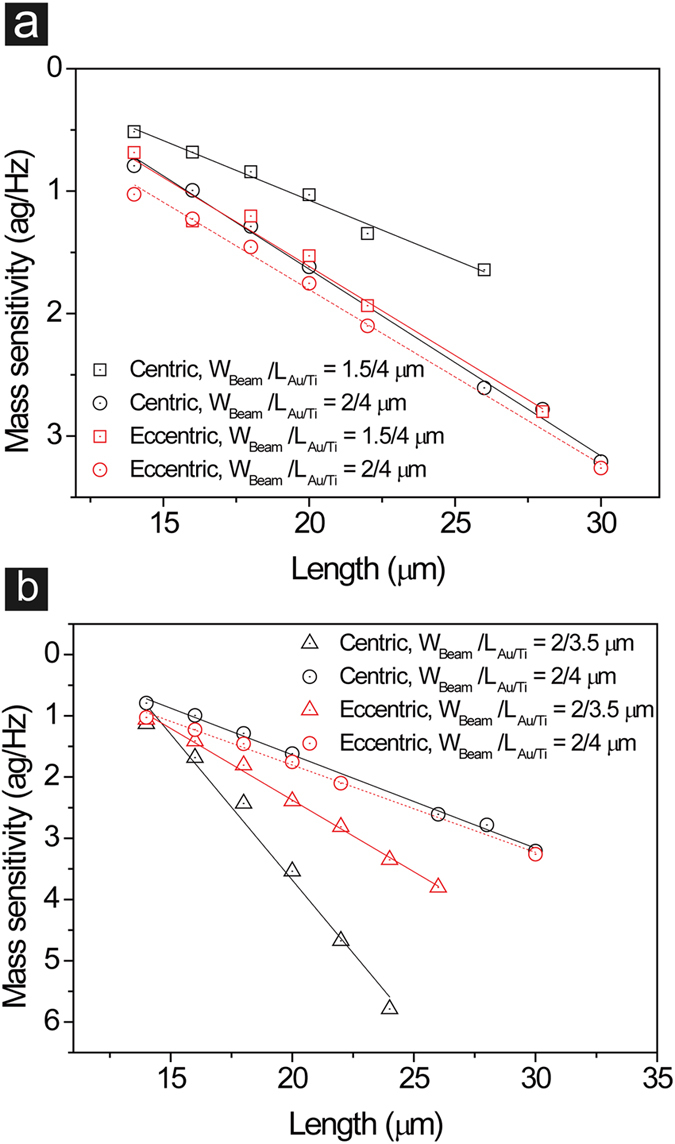
Effects of beam length, beam width (W_Beam_), loading position (centric and eccentric loading), and length of loaded mass (L_Au/Ti_) on the mass sensitivity of resonators. (**a**) Effects of changing beam width and loading position. (**b**) Effects of changing length of metal film and loading position (straight lines represent linear fits of data sets).

Using sequential mass loading, we investigated effects of different beam lengths (L_Beam_, 14–30 μm), beam widths (W_Beam_, 1.5 and 2 μm), loading positions (centric and eccentric loading), and lengths of metal films (L_Au/Ti_, 3.5 and 4 μm) on the mass sensitivity (Fig. 5). Metal deposition was limitedly carried out in the narrow region where the microbeam was less stressed at resonance. After each deposition, the resonant property was measured in linear and non-linear operating ranges. No significant change in operating characteristics was observed, suggesting that the effect of metal deposition on the physical property of the microbeam was negligible. For small masses, resonance frequency shifts showed linear relationship with masses. Therefore, the mass sensitivity of each resonator was determined by relating resonance frequency shift with sequential mass loading in the linear region (Figure S5 and Table S1). As expected, the mass sensitivity showed strong dependence on the beam length in all tested resonators (Fig. 5). Resonators with the narrower beam width (1.5 μm) showed higher mass sensitivity than those with the wider beam width (2 μm) due to decreased beam weight (Fig. 5a). Furthermore, centric loading showed higher mass sensitivity than eccentric loading (Fig. 5a), indicating that putting mass away from the center reduced the mass sensitivity. For 1.5 μm wide resonators, difference in the mass sensitivity between centric and eccentric loading decreased with the beam length since the distance between the center and the off-center became smaller as the beam length decreased (Figure S6). Previous studies also showed that mass sensitivity strongly depended on loading positions^31^,^32^,^33^. Asymmetrical mass loading on a beam can induce a shift of a nodal point, which causes oscillating motion to lose symmetry at the nodal point. This can lead to decrease in mass sensitivity due to decreases in peak displacement amplitude and vibration velocity of oscillation. The highest mass sensitivity was ~0.5 ag/Hz, which was achieved by the shortest and narrower microbeam using centric loading. These results suggest that higher mass sensitivity can be obtained by reducing the effective mass of the microbeam and loading the mass on the center.

In addition, the length of the metal film showed significant effect on the mass sensitivity (Fig. 5b). We expected that shorter distribution of the loaded mass would improve the mass sensitivity. However, loading the 4 μm long metal film showed higher mass sensitivity than the 3.5 μm long mass. This result may be attributed to local increase in stiffness of the microbeam by adding the metal film on the top surface. The longer metal film may result in greater increase in stiffness since it covers more area of the microbeam. It is interesting to note that eccentric loading resulted in higher mass sensitivity than centric loading when the 3.5 μm long metal film was loaded on the 2 μm wide microbeam (W_Beam_ = 2 μm, L_Au/Ti_ = 3.5 μm). This was opposite to the results in Fig. 5a, which were measured with 4 μm long masses. We speculate that when the length of metal film is reduced to 3.5 μm, increase in stiffness at the off-center may exert more beneficial effect on the mass sensitivity.

Mass sensitivity change per length, the slope from linear fit, was significantly affected by the loading position. For example, the highest mass sensitivity change per length was 0.477 ag/Hz·μm, which was measured from the 2 μm wide microbeam with 3.5 μm long mass loaded on the center (Table S2). By changing position of the mass to the off-center, the slope decreased to 0.234 ag/Hz·μm (Table S2), indicating that eccentric loading induced less dependence of the mass sensitivity on the beam length while exhibiting higher mass sensitivity than centric loading (Fig. 5b). However, assuming that the slope for centric loading can remain steeper than that for eccentric loading even when the beam length becomes much shorter than 14 μm, it is intriguing to speculate that the centric loading may result in higher mass sensitivity than the eccentric loading for those short microbeams. To maintain the steeper slope for centric loading, one may need to load a point-like mass for short microbeams instead of a mass with a finite length. Taken together, our results suggest that the mass sensitivity can be improved by reducing effective mass of a microbeam, loading a mass on the center, and using a point-like mass.

In particular, the mass sensitivity can be dramatically improved by decreasing the beam size to the nanoscale, which leads to decrease in the effective mass and increase in the resonance frequency. In addition, this change may result in increase in mass resolution. The minimum detectable mass (δm) can be estimated from the minimum measurable frequency variation (δf_0_) using δm = 2 m_eff_δf_0_/f_0_, where m_eff_ is the effective mass and f_0_ is the resonance frequency. To estimate frequency variation, resonance frequency of a resonator was repeatedly measured for 10 minutes (Figure S7). Extremely low frequency variation less than 5 Hz was observed for the resonator of L_Beam_ = 22 μm and m_eff_ = 1.08 × 10^−11^ g, corresponding to the minimum detectable mass of 7.04 × 10^−17^ g. Although the minimum detectable mass was ~ ag for micro-scale resonators in this study, we may achieve mass resolutions as low as ~ yg if the beam size can be successfully decreased to the nanoscale.

**Figure 6:**
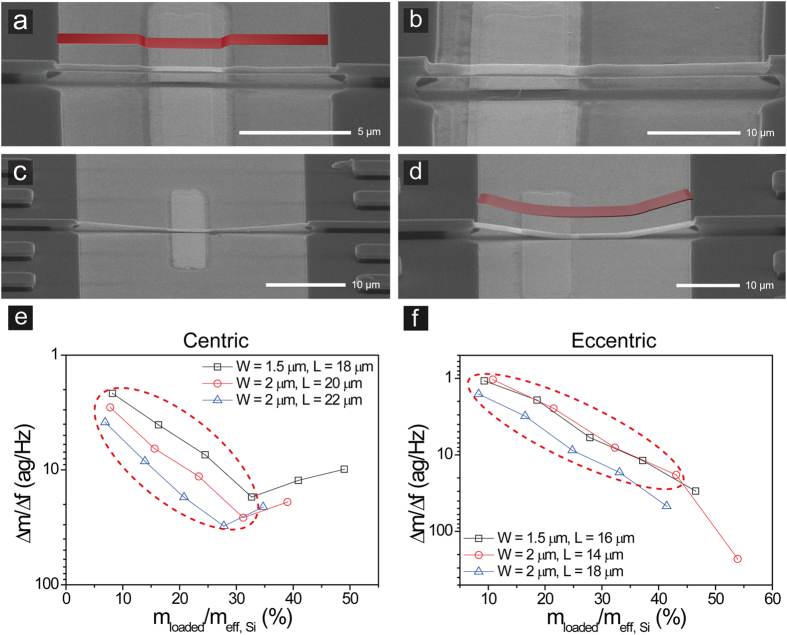
Local micro-buckling and global buckling of microbeams. SEM images of local micro-buckling for (**a**) centric and (**b**) eccentric loading. SEM images of global buckling for (**c**) centric and (**d**) eccentric loading. Changes in beam shape are schematically shown by insets (**a**,**d**). Effect of buckling on relationship between mass sensitivity and ratio of loaded mass to beam mass for (**e**) centric and (**f**) eccentric loading. Red dotted circles indicate that the properties of the microbeam are well maintained until the fourth mass loading.

Furthermore, we studied buckling behavior of microbeams caused by overloading mass, which significantly affects performance of the resonator. As sequential mass loading was continued, local strain caused by the mass induced micro-buckling only at the area where the metal film was deposited, while the remaining area maintained its shape (Fig. 6a and b). Further mass loading eventually caused global buckling of the microbeam, which has been well characterized by bending of entire beam structure (Fig. 6c and d). Two types of buckling were confirmed by SEM images showing distinct changes in microbeam shape. Both centric and eccentric loadings exhibited local micro-buckling, followed by global buckling. However, changes in resonant properties after buckling phenomena were dramatically different for two loading positions (Fig. 6e and f). For centric loading, the mass sensitivity exponentially decreased with the ratio of loaded mass to beam mass until the fourth loading. With the fifth loading, which induced the local micro-buckling, the mass sensitivity suddenly increased, indicating significant change in resonant property. The mass sensitivity fluctuated after the local micro-buckling, which made it difficult to predict the mass sensitivity based on exponential relationship with the mass ratio. After the sixth or seventh loading, measurement was no longer possible due to global buckling. In contrast, although eccentric loading showed similar exponential decrease in the mass sensitivity until the fourth loading, the local-micro buckling did not cause abrupt increase. Instead, the mass sensitivity remained close to its exponential relationship with the mass ratio or significantly decreased. Resonators eventually failed after the sixth or seventh loading due to global buckling. These results suggest that resonant property may be less susceptible to the local micro-buckling when the mass was loaded off center, and the loading position can influence dynamic range of mass sensor, accounting for failure caused by buckling.

Global buckling of a microbeam is known to be affected by beam length, beam thickness, amount of mass, and surface stiction^34^,^35^. Critical buckling stress, *σ*_*c*_, of a doubly-clamped beam is expressed as,


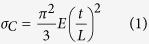


where *E* is the Young’s modulus, *t* is the beam thickness, and *L* is the beam length. Using *E* = 160 GPa, *t* = 100 nm, and *L* = 14–30 μm, the critical buckling stress of the resonator in the current study is 5.85 ~ 26.9 MPa. The internal stress, *σ*_*0*_, of a typical commercial SOI wafer is 10–100 MPa. Iwase *et al*. measured the internal stress of doubly-clamped Si microbeams fabricated on the SOI wafer to be 39 MPa^36^. When the internal stress is larger than the critical buckling stress, the critical buckling length (*l*_*c*_) can be estimated by,


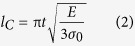


Using *σ*_*0*_ = 39 MPa, *E* = 160 GPa, and *t* = 100 nm, the critical buckling length is 11.6 μm, which is shorter than the beam length used in our study (14–30 μm), suggesting that the microbeams longer than the critical buckling length overcame the internal stress (Figure S8). This can be attributed to additional support from the SiN anchors, demonstrating that the SiN anchors, which improved the Q-factor (Fig. 2), can also increase the critical buckling length.

Micro-buckling reported in this study is characterized by a local bend at the border area, which lies between the metal-covered area and the bare microbeam surface (Figure S9). During the lift-off process for mass loading, the photoresist layer covers the microbeam surface except the area where the metal film will be deposited. If the photoresist layer can immobilize the microbeam structure and provide protection from strain, the bare area not covered with the photoresist is exposed to relatively larger strain. Since the photoresist typically form undercut profile for the lift-off, the undercut area of the microbeam surface is not covered with either photoresist or metal. When this bare area is exposed to strain from a loaded mass, it may become more susceptible to buckling due to absence of support from the photoresist layer, which can result in the local bend. Therefore, the micro-buckling may not be as significantly affected by the total beam length as the global buckling due to protection of the beam structure by the photoresist layer. Instead, the amount of mass may play a more important role. However, our study was limited to test step-wise increases in mass, and we were not able to quantify relationship between buckling and the amount of the mass with finer resolution.

## Conclusion

The present study demonstrated that the doubly-clamped Si microbeam resonator surrounded by the SiN anchors can achieve sub-ag/Hz mass sensitivity, which is extremely high for micro-scale MEMS mass sensors. To improve performance of the resonator, we have modified fabrication procedure to include deposition of the sacrificial SiN layer, which protected the Si microbeam surface, and to optimize the coverage ratio of the SiN anchors, which regulated oscillating motion. Resonant property is significantly improved by these changes. The highest Q-factor is ~30000, which is achieved by 1.5 μm wide microbeams with 56% coverage ratio. By characterizing how the mass sensitivity is affected by beam dimensions, loading position, and distribution of a loaded mass, we have suggested conditions for higher mass sensitivity, i.e., extremely low effective mass, a shorter and narrower symmetric beam structure, mass loading on the center (nodal point), and, ideally, loading a point mass. Furthermore, we identified two types of buckling, local micro- and global buckling, which were caused by different mechanisms. We also found that the SiN anchors increased the critical buckling length, and therefore, effectively prevented buckling since they provided additional support to the microbeam. Our results suggest that further miniaturization of the resonator based on the methods proposed in this study will achieve even higher mass sensitivity and enable precision measurement for various targets. For example, the detection of extremely small amount of environmental pollutions, such as hexavalent chromium (heavy metal) and geosmin (gas molecules), and bio-molecules.

## Methods

### Measurement system

Resonant properties of fabricated MEMS resonators were measured at a pressure between atmospheric pressure to ~1 mTorr at room temperature. The optical measurement system consisted of three-axis translation stage, electrostatic actuator, and Fabry-Perot interferometer. A doubly-clamped Si microbeam was actuated by the electric field between the microbeam and bottom electrode, which was generated by a function generator (Agilent 33250A, USA) using a frequency varying from 0.5 to 80 MHz and a voltage varying from 0.1 to 10 V_P-P_. A 17 mW He-Ne 633 nm laser (Thorlabs, USA) was used as a light source. Laser beam passed through a neutral-density filter before reaching the Si microbeam surface. Reflected beam from the microbeam surface passed through a beam splitter and was directed into a photodetector (Microsystems GmbH, Germany), which converts the intensity of the light to voltage signal. Then, the signal was amplified (MITEQ, USA) before it was measured by a lock-in amplifier (Stanford Research SR844, USA). In figures, the intensity of the incident light was represented by voltage amplitude of the phase-locked signal.

### Preparation of a substrate

Microbeam resonators were fabricated on a silicon-on-insulator (SOI) wafer consisting of a 1000 nm thick oxide layer buried underneath a 400 nm thick top Si (100) layer, which was lightly boron-doped (8.5–22 Ω cm). The top Si layer was thinned down to 100 nm by dry oxidation and wet etching. Thickness of the top Si was measured by an ellipsometer. Then, the top Si layer was doped by phosphorous ion implantation (dose = 3 × 10^13^ cm^−2^, ion energy = 8 keV), followed by rapid thermal annealing at 950 °C for 30 min in N_2_ environment. To protect the exposed Si beam surface during wet etching in heated phosphoric acid solution, a 15 nm thick SiN layer was deposited on the whole area of the wafer by LPCVD at 750 °C.

### Fabrication of a Si microbeam

Si microbeams with various widths (1.5 and 2 μm) and lengths (14 to 30 μm) were defined by photolithography using a 365 nm ultraviolet and dry etching. The SiN sacrificial layer and the top Si was dry etched by reactive ion etching using a mixed gas of CF_4_ and Ar at a pressure of 60 mTorr and a RF power of 100 W. The beam width was determined during this step but the beam length was separately determined during the following fabrication process for SiN anchors. Since the beam length was determined by the distance between the SiN anchors, we were able to minimize asymmetry of the Si microbeam and deformation of structure during wet etching. After fabricating the Si microbeam, the buried oxide layer was partially dry etched. Then, a 200 nm thick oxide hard mask layer was deposited on the whole area of the chip by LPCVD at 400 °C. To form the oxide hard mask for ion implantation, the oxide on the doping area was removed using photolithography and buffered oxide etchants (BOE) (6:1) for 120 sec. Then, the exposed SiN layer was etched in heated phosphoric acid solution.

### Control of coverage ratio

To form undercut structure, the buried oxide underneath the top Si was etched using time-controlled wet etching by BOE (6:1) at room temperature with spinning at 200 rpm. Then, samples with three different depths were fabricated. To fabricate the SiN anchor structure, a 260 nm thick SiN layer and a 491 nm thick oxide hard mask layer were deposited by LPCVD at 750 °C and 400 °C, respectively. The depths of fabricated samples were 269 nm, 420 nm and 721 nm, which were measured by SEM cross-sectional analysis.

## Additional Information

**How to cite this article:** Baek, I.-B. *et al*. Attogram mass sensing based on silicon microbeam resonators. *Sci. Rep.* **7**, 46660; doi: 10.1038/srep46660 (2017).

**Publisher's note:** Springer Nature remains neutral with regard to jurisdictional claims in published maps and institutional affiliations.

## Supplementary Material

Supplementary Information

## Figures and Tables

**Table 1:** Dimensions of microbeams (width × length), their coverage ratios, and highest Q-factors measured from each sample (A, B, C).

Beam width (μm)	1.5			2		
Beam length (μm)	12–28			12–28		
Sample	A	B	C	A	B	C
Coverage ratio (%)	35.9	56	96.1	26.9	42	72.1
Q-factor (max)	6070	30131	5616	5616	29134	14688

*Coverage ratio (%) = ((2 × b)/a) × 100, a = W_Beam_, b = W_Undercut_.

**Table 2:** Changes in resonant properties by sequential mass loading (*f*_*0*_: resonance frequency, *Δf*: full width at half maximum, *V*_*P*-*P*_: actuation voltage).

Deposition	None	1^st^	2^nd^	3^rd^	4^th^	5^th^
*Mass* (g)		5.31 × 10^−13^	1.06 × 10^−12^	1.59 × 10^−12^	2.13 × 10^−12^	2.66 × 10^−12^
*f*_*0*_ (MHz)	5.35	5.04	4.74	4.49	4.33	4.13
*Δf* (Hz)	300	310	370	340	350	360
*V*_*P*-*P*_ (V)	0.55	0.6	0.65	0.75	0.7	1.8^*^
Q-factor	17847	16793	12805	13201	12382	11467

*Sudden increase in actuation voltage.

## References

[b1] Stowe T. D. . Attonewton force detection using ultrathin silicon cantilevers. Appl. Phys. Lett. 71, 288–290, doi: 10.1063/1.119522 (1997).

[b2] Naik A. K. Hanay M. S., Hiebert W. K., Feng X. L. & Roukes M. L. Towards single-molecule nanomechanical mass spectrometry. Nat. Nanotech. 4, 445–450, doi: 10.1038/nnano.209.152 (2009). PMC3846395 19581898

[b3] Hajjam A. & Pourkamali S. Fabrication and Characterization of MEMS-Based Resonant Organic Gas Sensors. IEEE Sens. J. 12, 1958–1964 (2012).

[b4] Dennis J.-O., Ahmed A.-Y. & Khir M.-H. Fabrication and Characterization of a CMOS-MEMS Humidity Sensor. Sensors. 15, 16674–16687, doi: 10.3390/s150716674 (2015). 26184204 PMC4541900

[b5] Zhang X. C., Myers E. B., Sader J. E. & Roukes M. L. Nanomechanical Torsional Resonators for Frequency-Shift Infrared Thermal Sensing. Nano Lett. 13, 1528–1534, doi: 10.1021/nl304687p (2013). 23458733

[b6] Gupta A. K. . Anomalous Resonance in a Nanomechanical Biosensor. PNAS. 103, 13362–13367, doi: 10.1073/pnas.0602022103 (2006). 16938886 PMC1569169

[b7] Yang Y. T., Callegari C., Feng X. L., Ekinci K. L. & Roukes M. L. Zeptogram-Scale Nanomechanical Mass Sensing. Nano Lett. 6, 583–586, doi: 10.1021/nl052134m (2006). 16608248

[b8] Llic B. . Attogram detection using nanoelectromechanical oscillators. J. Appl. Phys. 95, 3694–3703, doi: 10.1063/1.1650542 (2004).

[b9] Verbridge S. S., Parpia J. M., Reichenbach R. B., Bellan L. M. & Craighead H. G. High quality factor resonance at Room temperature with nanostrings under high tensile stress. J. Appl. Phys. 99, 124304, doi: 10.1063/1.2204829 (2006).

[b10] Cullinan M. A., Panas R. M., DiBiasio C. M. & Culpepper M. L. Scaling electromechanical sensors down to the nanoscale. Sens. Actuators A. 187, 162–173, doi: 10.1016/j.sna.2012.08.035 (2012).

[b11] Gill J. J.-Y., Ngo L. V., Nelson P. R. & Kim C.-J. Elimination of Extra Spring Effect at the Step-Up Anchor of Surface-Micromachined Structure. J. Microelectromech. Syst. 7, 114–121, doi: 10.1109/84.661393 (1998).

[b12] Mohanty P., Harrington D. A., Ekinci K. L., Yang Y. T., Murphy M. J. & Roukes M. L. Intrinsic dissipation in high-Frequency micromechanical resonators. Phys. Rev. B. 66, 085416, doi: 10.1103/PhysRevB.66.085416 (2002).

[b13] Mihailovich R. E. & MacDonald N. C. Dissipation measurements of vacuum-operated single-crystal silicon microresonators. Sens. Actuators A. 50, 199–207, doi: 10.1016/0924-4247(95)0180-7 (1995).

[b14] Frangi A., Cremonesi M., Jaakkola A. & Pensala T. Analysis of anchor and interface losses in piezoelectric MEMS resonators. Sens. Actuators A. 190, 127–135, doi: 10.1016/j.sna.2012.10.022 (2013).

[b15] Lee J. E.-Y., Yan J. & Seshia A. A. Study of lateral mode SOI-MEMS resonators for reduced anchor loss. J. Micromech. Microeng. 21, 045010, doi: 10.1088/0960-1317/21/4/045010 (2011).

[b16] Fang W. & Wickert J. A. Determining mean and gradient residual stress in thin films using micromachined cantilevers. J. Micromech. Microeng. 6, 301–309, doi: 10.1088/0960-1317/6/002 (1996).

[b17] Mathew J. P. . Nanoscale Electromechanics To Measure Thermal Conductivity, Expansion, and Interfacial Losses. Nano Lett. 15, 7621–7626, doi: 10.1021/acs.nanolett.5b03451 (2015). 26479952

[b18] Postma H. W. Ch., Kozinsky I., Husain A. & Roukes M. L. Dynamic range of nanotube- and nanowire-based electromechanical systems. Appl. Phys. Lett. 86, 223105, doi: 10.1063/1.1929098 (2005).

[b19] Ekinci K. L. & Roukes M. L. Nanoelectromechanical Systems. Rev. Sci. Instrum. 76, 061101, doi: 10.1063/1.1927327 (2005).

[b20] Azak N. O. . Nanomechanical displacement detection using fiber-optic interferometry. Appl. Phys. Lett. 91, 093112, doi: 10.1063/1.2776981 (2007).

[b21] Petitgrand S., Courbet B. & Bosseboeuf A. Characterization of static and dynamic optical actuation of Al microbeams by microscopic interferometry techniques. J. Micromech. Microeng. 13, S113–S118, doi: 10.1088/0960-1317/13/4/319 (2003).

[b22] Karabacak D., Kouh T. & Ekinci K. L. Analysis of optical interferometric displacement detection in nanoelectromechanical systems. J. Appl. Phys. 98, 124309, doi: 10.1063/1.2148630 (2005).

[b23] Ekinci K. L., Yang Y. T., Huang M. H. & Rukes M. L. Balanced electronic detection of displacement in nanoelectromechanical systems. Appl. Phys. Lett. 81, 2253–2255, doi: 10.1063/1.1507833 (2002).

[b24] Camassel J., Falkovsky L. A. & Planes N. Strain effect in silicon-on-insulator materials: Investigation with optical phonons. Phys. Rev B. 63, 035309, doi: 10.1103/PhysRevB.63.035309 (2000).

[b25] Bifano T. G., Johnson H. T., Bierden P. & Mali R. K. Elimination of stress-induced curvature in thin-film structures. J. Microelectromech. Syst. 11, 592–597, doi: 10.1109/JMEMS.2002.802908 (2002).

[b26] Somà A. & Ballestra A. Residual stress measurement method in MEMS microbeams using frequency shift data. J. Micromech. Microeng. 19, 095023, doi: 10.1088/0960-1317/19/9/095023 (2009).

[b27] Guyot N., Harmand Y. & Mézin A. The role of the sample shape and size on the internal stress induced curvature of thin-film substrate systems. Int. J. Solids. Struct. 41, 5143–5154, doi: 10.1016/j.ijsolstr.2004.03.015 (2004).

[b28] Fang W. & Wickert J. A. Post buckling of micromachined beams. J. Micromech. Microeng. 4, 116–122, doi: 10.1088/0960-1317/4/3/004 (1994).

[b29] Wang S., Song J., Kim D.-H., Huang Y. & Rogers J. A. Local versus global buckling of thin films on elastomeric substrates. Appl. Phys. Lett. 93, 023126, doi: 10.1063/1.2956402 (2008).

[b30] Baek I.-B. . The control of oscillation mode in silicon microbeams using silicon nitride anchor. App. Phys. Lett. 105, 103101, doi: 10.1063/1.4895119 (2014).

[b31] Zhang K., Chai Y. & Fu J. Study of node and mass sensitivity of resonant mode based cantilevers with concentrated mass loading. AIP Adv. 5, 127109, doi: 10.1063/1.4937741 (2015).

[b32] Zhang K., Chai Y. & Cheng Z.-Y. Location Dependence of Mass Sensitivity for Acoustic Wave Devices. Sensors. 15, 24585–24594, doi: 10.3390/s150924585 (2015). 26404313 PMC4610577

[b33] Yi J. W., Shih W. Y. & Shih W.-H. Effect of length, width, and mode on the mass detection sensitivity of piezoelectric unimorph cantilevers. J. Appl. Phys. 91, 1680–1686, doi: 10.1063/1.1427403 (2002).

[b34] Tas N., Sonnenberg T., Jansen H., Legtenberg R. & Elwenspoek M. Stiction in surface micromachining. J. Micromech. Microeng. 6, 385–397, doi: 10.1088/0960-1317/6/4/005 (1996).

[b35] Legtenberg R., Tilmans H. A. C.; Elders J. & Elwenspoek M. Stiction of surface micromachined structure after rinsing and drying: model and investigation of adhesion mechanisms. Sens. Actuators A. 43, 230–238, doi: 10.1016/0924-4247(93)00654-M (1994).

[b36] Iwase E. . Control of buckling in large micromembranes using engineered support structures. J. Micromech. Microeng. 22, 065028, doi: 10.1088/0960-1317/22/6/065028 (2012).

