# Supplementary material for: Attogram mass sensing based on silicon microbeam resonators
Source: Sci Rep. 2017 Apr 21;7:46660. doi: 10.1038/srep46660 (PMC5399360; doi:10.1038/srep46660)
Supplement: Supplementary Information [file srep46660-s1.doc]

***Supplementary Information***

Attogram mass sensing based on silicon microbeam resonators

**In-Bok Baek^1, 2^, Sangwon Byun^2^, Bong Kuk Lee^2^, Jin-Hwa Ryu^2^, Yarkyeon Kim^2^, Yong Sun Yoon^2^, Won Ik Jang^2^, Seongjae Lee^1,*^, and Han Young Yu^2,*^**

^1^ Department of Physics, Research Institute for Natural Sciences, Hanyang University, 222 Wangsimri-ro, Seongdonggu, Seoul, 04763, Korea|.

^2^ Bio-Medical IT Convergence Research Department, Electronics and Telecommunications Research Institute (ETRI), 218 Gajeongno, Yuseong, Daejeon, 34129, Korea.

^*^corresponding. [uhan0@etri.re.kr](mailto:uhan0@etri.re.kr)

| **Centric loading** | | | | | |
| --- | --- | --- | --- | --- | --- |
| W_Beam_ | 1.5 µm | W_Beam_ | 2 µm | | |
| L_Au/Ti_ | 4 µm | L_Au/Ti_ | 3.5 µm | L_Au/Ti_ | 4 µm |
| Length  (µm) | Mass sensitivity  (ag/Hz) | Length  (µm) | Mass sensitivity  (ag/Hz) | Length  (µm) | Mass sensitivity  (ag/Hz) |
| 14 | 0.51 | 14 | 1.13 | 14 | 0.79 |
| 16 | 0.68 | 16 | 1.68 | 16 | 0.99 |
| 18 | 0.84 | 18 | 2.43 | 18 | 1.29 |
| 20 | 1.03 | 20 | 3.54 | 20 | 1.62 |
| 22 | 1.35 | 22 | 4.67 | 26 | 2.61 |
| 26 | 1.64 | 24 | 5.79 | 28 | 2.78 |
|  | | | | 30 | 3.21 |

| **Eccentric loading** | | | | | |
| --- | --- | --- | --- | --- | --- |
| W_Beam_ | 1.5 µm | W_Beam_ | 2 µm | | |
| L_Au/Ti_ | 4 µm | L_Au/Ti_ | 3 µm | L_Au/Ti_ | 4 µm |
| Length  (µm) | Mass sensitivity  (ag/Hz) | Length  (µm) | Mass sensitivity  (ag/Hz) | Length  (µm) | Mass sensitivity  (ag/Hz) |
| 14 | 0.69 | 14 | 1.06 | 14 | 1.03 |
| 16 | 1.24 | 16 | 1.41 | 16 | 1.23 |
| 18 | 1.20 | 18 | 1.81 | 18 | 1.46 |
| 20 | 1.53 | 20 | 2.39 | 20 | 1.75 |
| 22 | 1.94 | 22 | 2.81 | 22 | 2.10 |
| 28 | 2.80 | 24 | 3.35 | 30 | 3.26 |
|  | | 26 | 3.80 |  | |
| * W_Beam_ = Si microbeam width, L_Au/Ti_ = Au/Ti film length. | | | | | |
|  | | | | | |

**Table S1.** Summary of the mass sensitivity from data sets in Figure S5.

| **Centric loading** | | | |
| --- | --- | --- | --- |
| W_Beam_ / L_Au/Ti_ (µm) | 1.5 / 4 | 2 / 3.5 | 2 / 4 |
| Mass sensitivity  per length (ag/Hz· µm) | 0.097 | 0.477 | 0.153 |
|  |  |  |  |
| **Eccentric loading** | | | |
| W_Beam_ / L_Au/Ti_ (µm) | 1.5 / 4 | 2 / 3.5 | 2 / 4 |
| Mass sensitivity  per length (ag/Hz· µm) | 0.145 | 0.234 | 0.143 |

**Table S2.** Mass sensitivity change per length estimated from data sets in Figure 5.


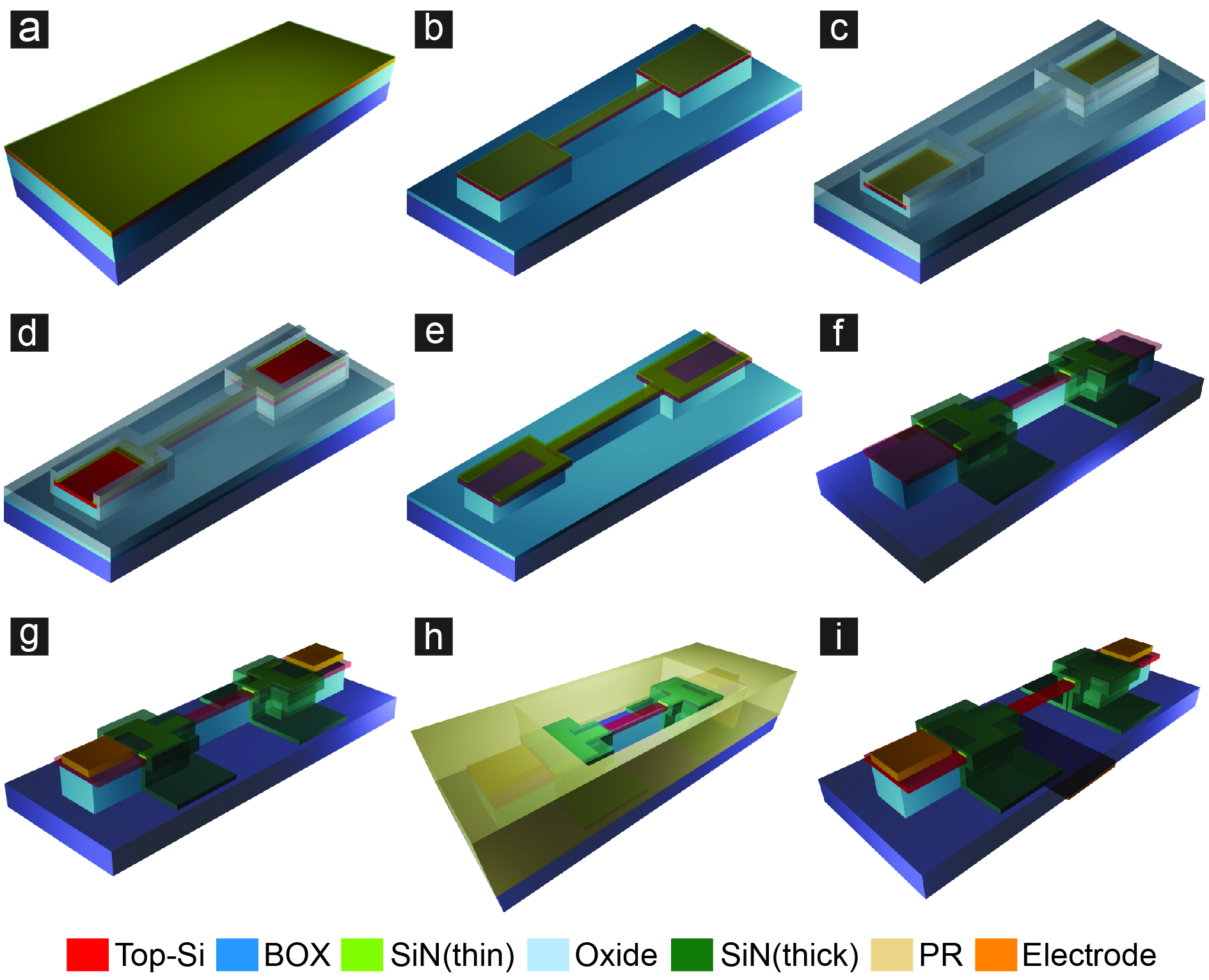


**Figure S1.** Schematic diagrams of fabrication processes for a doubly-clamped Si microbeam resonator with a SiN sacrificial layer. (a) Deposition of a 15 nm thick SiN sacrificial layer on the top Si after thining and ionimplantation process. (b) Dry etching of the top Si and buried oxide. (c) Wet etching of silicon dioxide hard mask for contact area open. (d) Wet etching of the SiN sacrificial layer for ionimplantation of contact pads. (e) Wet etching of the buried oxide under the top Si. (f) Wet etching of SiN anchors using the silicon dioxide hard mask. (g) Electrode deposition for electrical contacts (Au/Ni electrode evaporation and lift-off process). (h) Opening of buried oxide region for selective etching. (i) The fabricated doubly-clamped Si microbeam resonator surrounded by the SiN anchors.


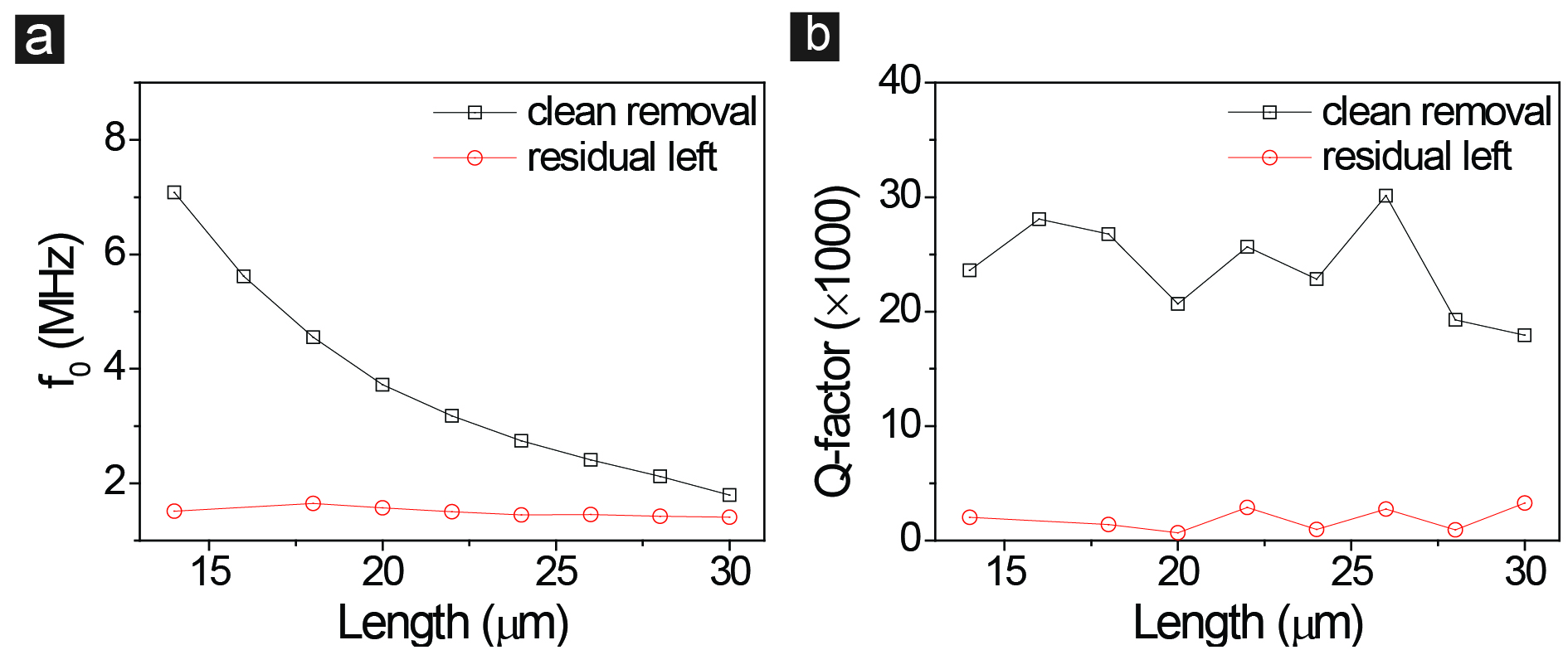


**Figure S2**. Effect of the residual SiN sacrificial layer left on microbeam surface on (a) resonance frequency and (b) Q-factor. Resonators with the residual show significantly lower resonance frequency and Q-factor than those without the residual. Decreases in resonance frequency and Q-factor are caused by non-uniformly distributed local residual and damaged microbeam surface. The beam width is 1.5 µm and the beam length is varied from 14 to 30 µm.


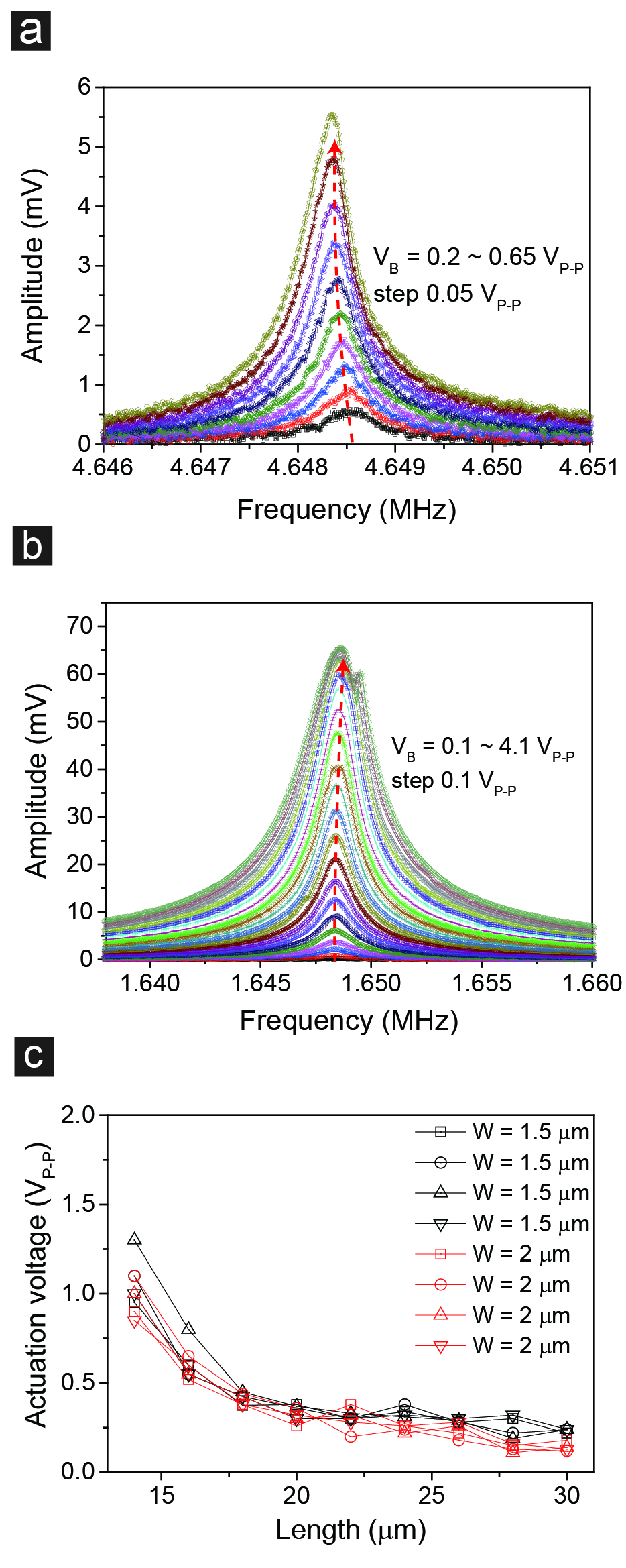


**Figure S3.** Effect of the residual SiN sacrificial layer left on microbeam surface on resonant properties. Resonators with the same beam dimensions are tested (length = 18 µm, width = 2 µm, thickness = 100 nm). Although resonators with the residual SiN sacrificial layer show higher linearity, their actuation voltage is significantly increased. (a) Linearity of oscillating motion of a microbeam without the residual. Actuation voltage is varied from 0.2 to 0.65 V_P-P_ with a 0.05 V_P-P_ step. (b) Linearity of oscillating motion of a microbeam with the residual. Actuation voltage is varied from 0.1 to 4.1 V_P-P_ with a 0.1 V_P-P_ step. (c) Actuation voltage versus beam length for resonators without the residual SiN sacrificial layer. The beam width is 1.5 µm and the beam length is varied from 14 to 30 µm.


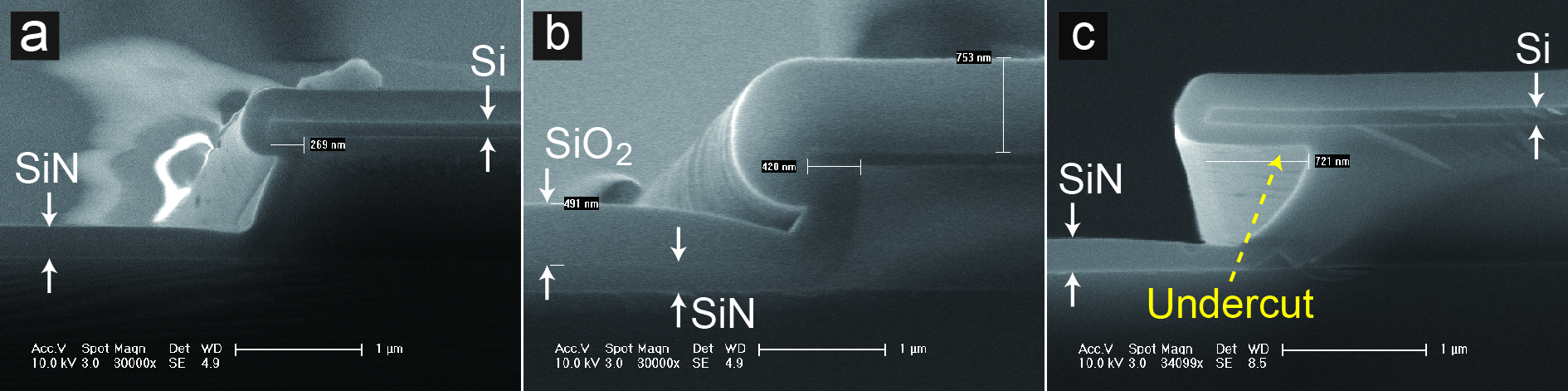


**Figure S4.** Cross-sectional SEM images of Si microbeams and undercut areas covered by SiN layers. The undercut area was formed by time-control wet etching (a = 269 nm, b = 420 nm, c = 721 nm). Anchor structure was fabricated by depositing a 260 nm thick SiN layer on both undercut area and Si microbeam. (a, b) The structure consists of a 491 nm thick oxide hard mask layer and a 260 nm thick SiN layer on the Si microbeam. (c) The SEM image shows bent-up of the Si microbeam at the undercut area.


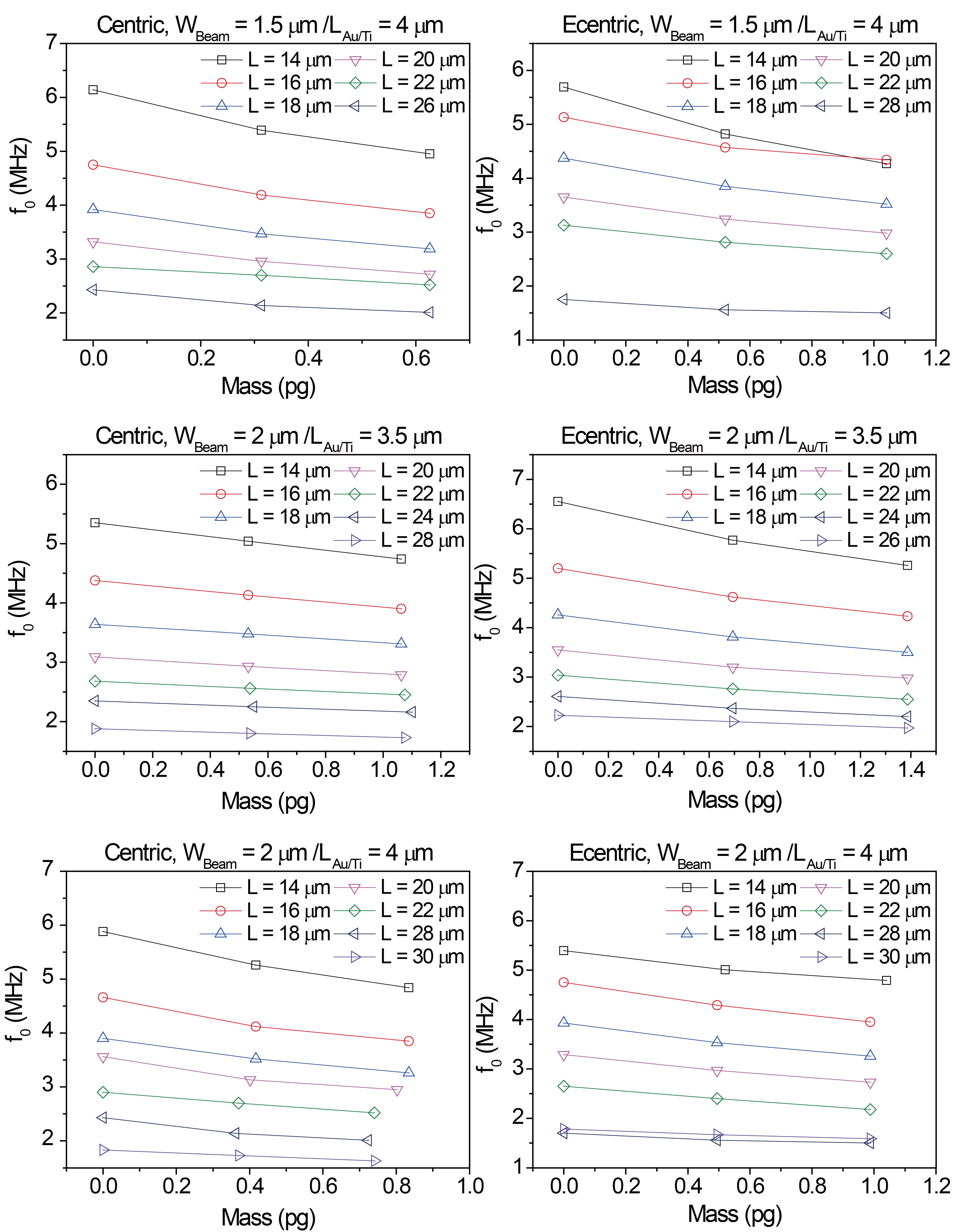


**Figure S5.** Shift in resonance frequency relative to sequential mass loading with varying beam lengths. Two different loading positions, center (left panels) and off-center (right panels), are tested. Estimated values of mass sensitivity are summarized in Table S1.


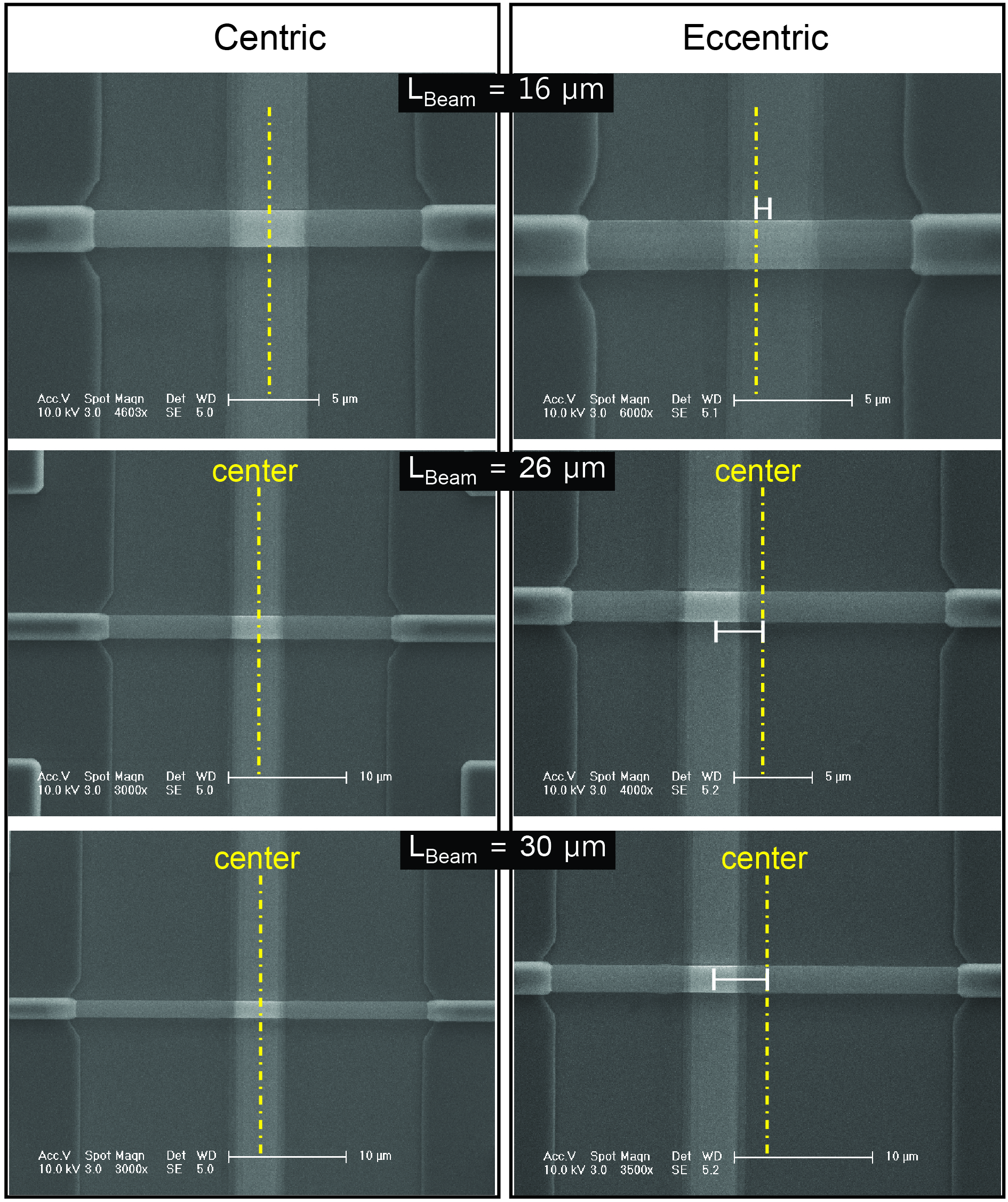


**Figure S6.** SEM images showing distance between positions of centric (left panels) and eccentric (right panels) loading with varying beam lengths. The position of eccentric loading moves closer to the center as the beam length decreases. As a result, difference in mass sensitivity between centric and eccentric loading decreases with the beam length.


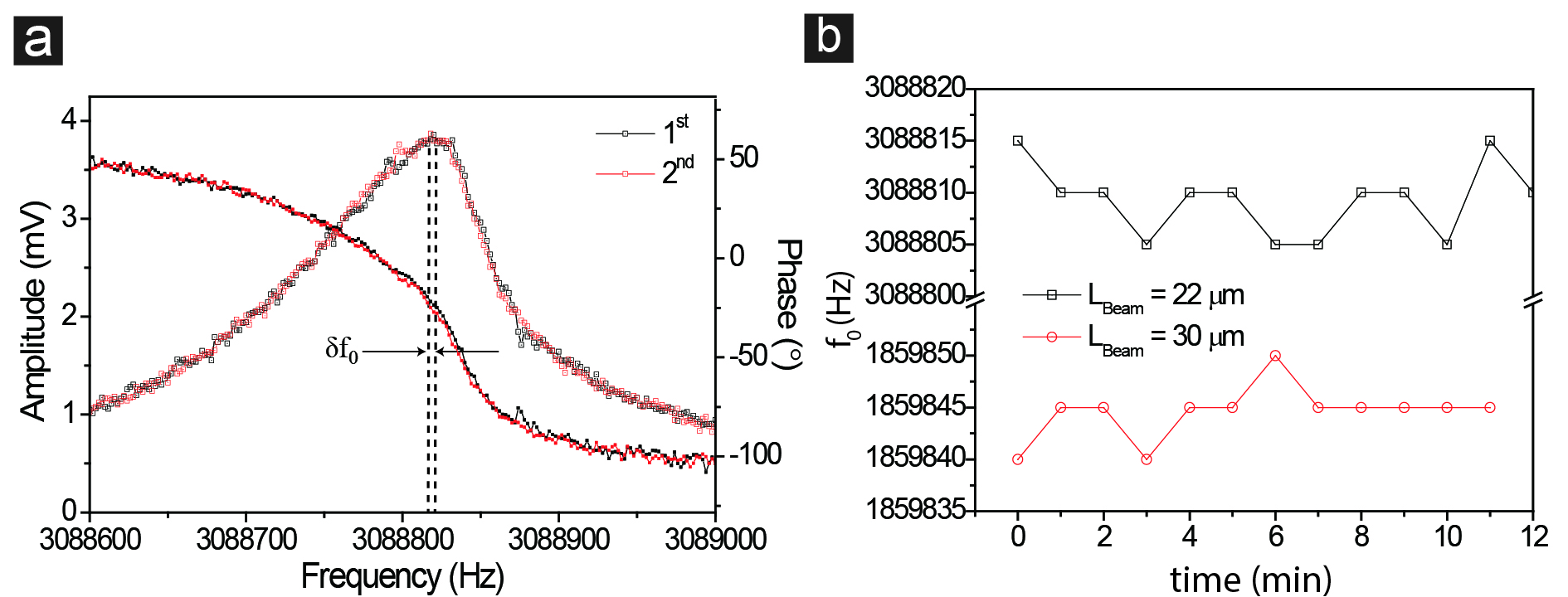


**Figure S7.** Estimating limit of detection. (a) To track change in resonance frequency, frequency response of a resonator is repeatedly measured at 1 min interval for 10 min at room temperature. As an example, frequency responses from the first and the second measurements of the 22 µm long, 2 µm wide, and 100 nm thick microbeam are shown. Difference in the resonance frequency between the first and the second trial is indicated by δf_0_. (b) Resonance frequency (f_0_) versus time for resonators with two different beam lengths, 22 µm and 30 µm. Both resonators show extremely low variation in resonance frequency. Standard deviations in resonance frequency were 3.4 Hz and 2.5 Hz for L_Beam_ = 22 µm (f_0_ = 3.08809 MHz) and L_Beam_ = 30 µm (f_0_ = 1.859844 MHz), respectively, which can be converted to ± 1.6 and ± 2.7 ppm (parts per million).


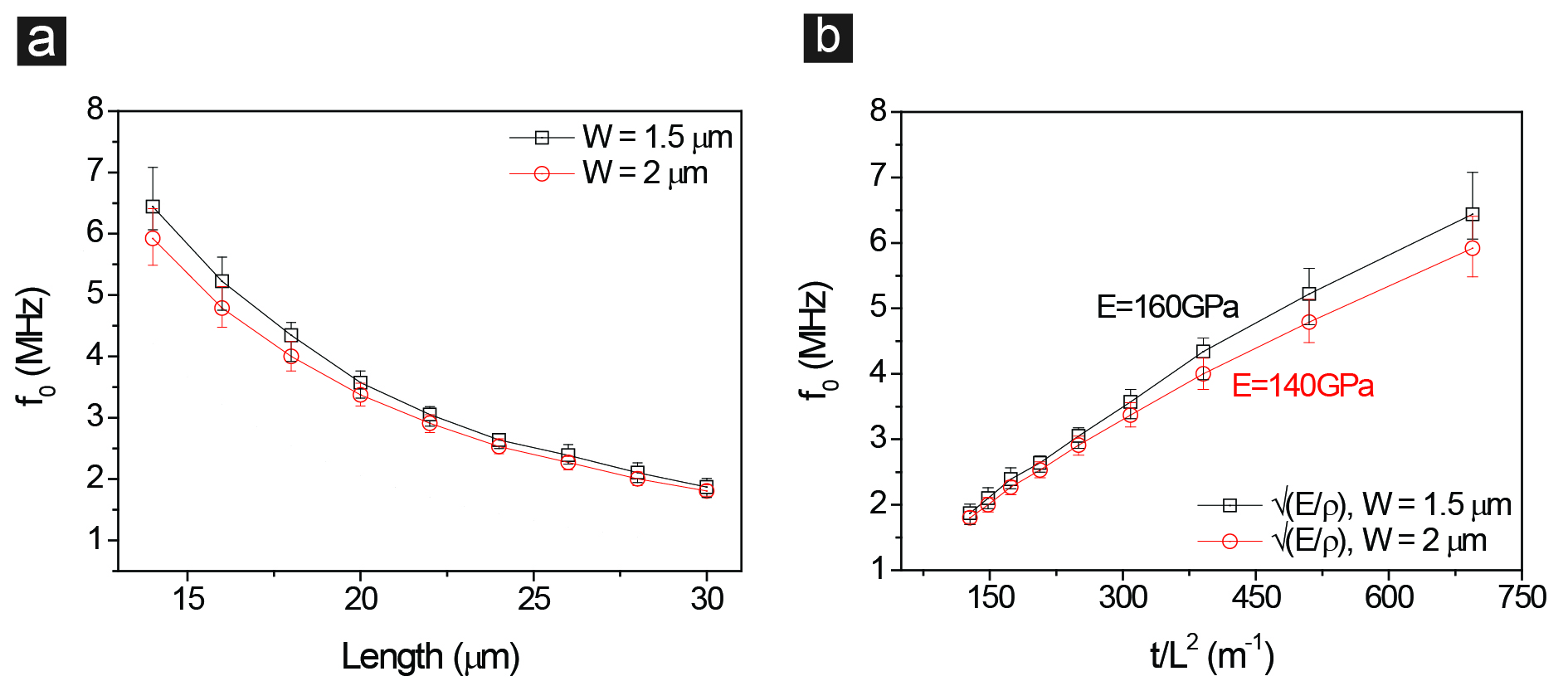


**Figure S8.** (a) Dependence of resonance frequency on beam length. The beam length is varied from 14 to 29 µm, and the beam widths are 1.5 µm and 2 µm. (b) Estimation of Young’s modulus from the data shown in a.


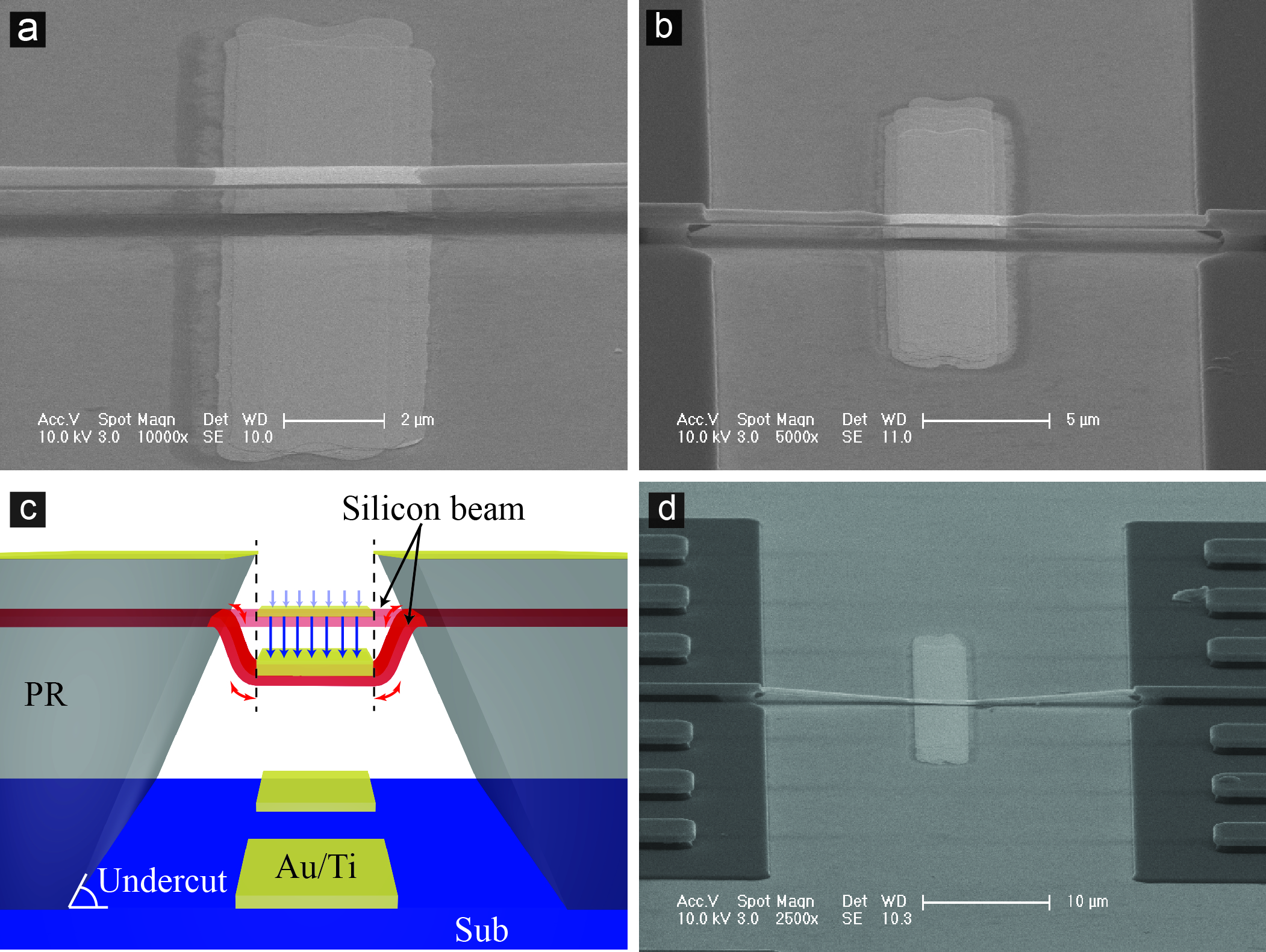


**Figure S9.** SEM images of microbeam buckling caused by sequential mass loading on the center. (a) A normal microbeam after the third mass loading. (b) Local micro-buckling after the fifth mass loading. (c) Schematic illustration of local micro-buckling showing how photoresist layer and undercut area affect local strain on the microbeam. (d) Global buckling after the sixth mass loading.
